# An improvement of ComiR algorithm for microRNA target prediction by exploiting coding region sequences of mRNAs

**DOI:** 10.1186/s12859-020-3519-5

**Published:** 2020-09-16

**Authors:** Giorgio Bertolazzi, Panayiotis V. Benos, Michele Tumminello, Claudia Coronnello

**Affiliations:** 1 Department of Economics, Business and Statistics, University of Palermo, Palermo, Italy; 2 Advanced Data Analysis Group, Fondazione Ri.MED, Palermo, Italy; 3 Department of Computational and Systems Biology, University of Pittsburgh, Pittsburgh, USA

**Keywords:** microRNA target prediction, 3’UTR, Coding region, AGO1, *Drosophila melanogaster*

## Abstract

MicroRNA are small non-coding RNAs that post-transcriptionally regulate the expression levels of messenger RNAs. MicroRNA regulation activity depends on the recognition of binding sites located on mRNA molecules. ComiR is a web tool realized to predict the targets of a set of microRNAs, starting from their expression profile. ComiR was trained with the information regarding binding sites in the 3’utr region, by using a reliable dataset containing the targets of endogenously expressed microRNA in *D. melanogaster* S2 cells. This dataset was obtained by comparing the results from two different experimental approaches, i.e., inhibition, and immunoprecipitation of the AGO1 protein--a component of the microRNA induced silencing complex.

In this work, we tested whether including coding region binding sites in ComiR algorithm improves the performance of the tool in predicting microRNA targets. We focused the analysis on the *D. melanogaster* species and updated the ComiR underlying database with the currently available releases of mRNA and microRNA sequences. As a result, we find that ComiR algorithm trained with the information related to the coding regions is more efficient in predicting the microRNA targets, with respect to the algorithm trained with 3’utr information. On the other hand, we show that 3’utr based predictions can be seen as complementary to the coding region based predictions, which suggests that both predictions, from 3’utr and coding regions, should be considered in comprehensive analysis.

Furthermore, we observed that the lists of targets obtained by analyzing data from one experimental approach only, that is, inhibition or immunoprecipitation of AGO1, are not reliable enough to test the performance of our microRNA target prediction algorithm. Further analysis will be conducted to investigate the effectiveness of the tool with data from other species, provided that validated datasets, as obtained from the comparison of RISC proteins inhibition and immunoprecipitation experiments, will be available for the same samples. Finally, we propose to upgrade the existing ComiR web-tool by including the coding region based trained model, available together with the 3’utr based one.

## Background

MicroRNA genes (miRNAs) are small non-coding RNAs that post - transcriptionally regulate the expression level of messenger RNAs (mRNAs). MicroRNAs are critical in many important biological processes, and are important markers for many diseases. A miRNA can bind many mRNAs and an mRNA can be bound by several miRNAs. The ability of predicting the targets of the endogenous miRNAs is then crucial to understand the processes they are involved in.

MicroRNA regulation activity depends on the recognition of binding sites located on mRNA molecules. MicroRNA target prediction algorithms are generally based on Watson-Crick base-pair matching [1–3]. Few other methods use the miRNA expression profile as additional information, namely, GenMir++ [4], PicTar [5], Talasso [6]. Recently, we introduced an innovative algorithm to predict targets of endogenous miRNAs, named ComiR (Combinatorial miRNA targeting) [7, 8]. ComiR incorporates miRNA expression in a thermodynamic binding model, and it associates each gene with the probability of being a target of a set of miRNAs.

The miRNA targets identification has been mainly based on the search of mRNA binding sites contained in the 3’utr region [9]. It is also known that miRNAs bind the coding region [10], and our previous results [11] showed that the coding region plays a role in distinguishing RISC machinery targets. Therefore, the information contained in the coding region can’t be ignored for the miRNA target prediction.

**Fig. 1:**
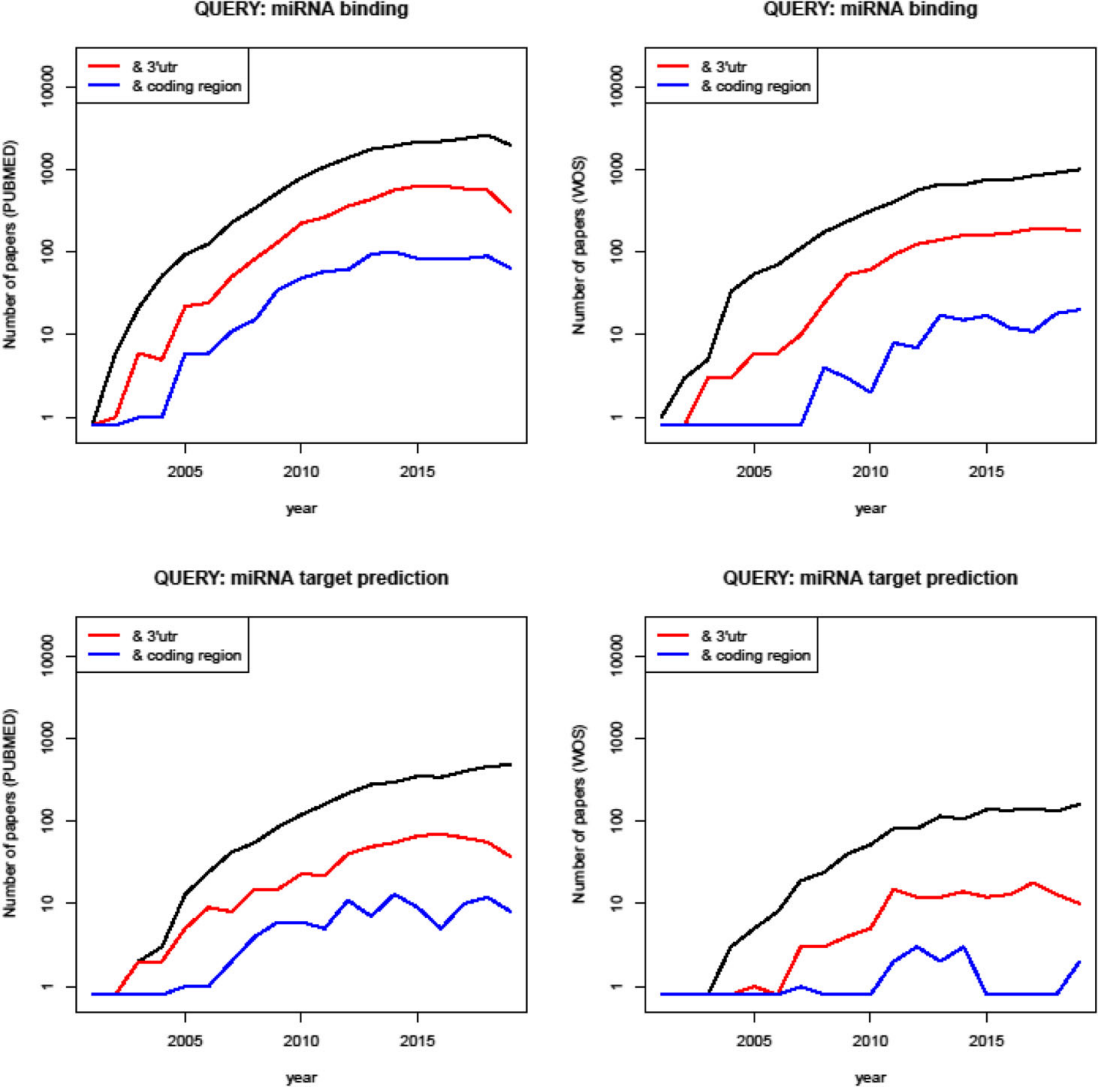
Quantification of scientific production regarding miRNA topics, updated to Jan 2020. Left panels concern queries to the PUBMED repository, while right panels concern queries to the ISI Web of Science repository. Black lines indicate the temporal evolution of the number of papers found through the main query, which is indicated in the title of each panel. Red and blue lines indicate the temporal evolution of the number of papers found by combining the main query with the words “3’utr” and “coding region”, respectively

Figure 1 reports the number of outcomes of four queries to PUBCHEM and ISI Web of Science repositories, namely, the number of papers associated with the joint queries 1) “miRNA target prediction” & “3’UTR”; 2) “miRNA target prediction” & “coding region”; 3) “miRNA binding” & “3’UTR”; and 4) “miRNA binding” & “coding region”. It’s worth to note that, despite continuous evidences of the presence of binding sites in the mRNA coding region, the incidence of the word “3’UTR” is steadily one order of magnitude higher than the one of “coding region”. Indeed, we hypothesize that words “miRNA” and “3’UTR” have been linked together since the discovery of microRNAs, whereas the association between “miRNA” and “coding region” is less explored. The focus of the actual version of ComiR on binding sites in the 3’UTR only is a typical example.

In this study, we propose to upgrade the ComiR algorithm, by introducing information about the binding sites contained in the coding region of the genes. We show that the information contained in the coding region significantly improves the accuracy of ComiR predictions.

## Methods

ComiR is a user friendly web tool described in [8]. The user has to provide a list of miRNAs and their expression levels. The output is a ranked vector of scores; therefore, each gene is associated with a reliability of being a target of the set of miRNAs given in input. The original algorithm contains a Support Vector Machine (SVM) based algorithm that incorporates the miRNA target prediction results of four individual tools (i.e. PITA [12], miRanda [13] and TargetScan [14] and miRSVR [15]) in 3’UTR. Due to a break in maintenance of mirSVR scores, in this work, we will only consider the PITA, miRanda and TargetScan predictions.

This work is focused on *Drosophila melanogaster* (Dme) miRNA target prediction. The dataset used to train and test the algorithm is derived by the combination of data from two experiments: Ago1 immunoprecipitation (IP) [16] and Ago1 depletion [17], both performed in *D. melanogaster* S2 cells. We distinguish between four sets of genes: set I, 152 genes enriched in AGO1 IP and upregulated after AGO1 depletion; set II, 1039 genes enriched in AGO1 IP and not upregulated after AGO1 depletion; set III, 300 genes not enriched in AGO1 IP and upregulated in AGO1 depletion; set IV, 5509 genes not enriched in AGO1 IP and not upregulated in AGO1 depletion.

We downloaded the 3’UTR and coding region sequences of genes in *D. melanogaster* species from Ensembl/bioMart (release BDGP6.22). We only considered Dme genes with annotated both the 3’UTR and coding region. Consequently, our final dataset was composed by 139 genes in set I, 929 genes in set II, 253 genes in set III and 4738 genes in set IV.

The dataset also contains the expression profile of 28 top miRNAs in S2 cells. The whole set of 469 mature miRNA sequences were downloaded from miRBase (release 22). For each miRNA, we applied the PITA, miRanda and TargetScan algorithms, in order to detect the binding sites in both the 3’UTR and the coding region of each gene. These predictions, integrated with the opportune miRNA expression profile, have been used to compute the scores used to feed the SVM.

The performance of ComiR algorithm has been evaluated by implementing leave-one-out Cross-Validation (LOOCV) procedure (one by one, each gene is left out from the training set at each step of the procedure) and by using a test set independent of the training set. The comparison between two ROC curves is performed with the DeLong test [18]. Two empirical cumulative distribution functions (ECDF) are compared by applying the Wilcoxon test. *P*-values lower than 0.01 have been considered as statistically significant.

## Results

Similarly to the original version of ComiR, the SVM has been trained with set I (139 genes) as positive set and the 139 most highly expressed genes from set IV as negative set (named top-set-IV). To evaluate which part of the gene sequence produce the best prediction accuracy, we have implemented a SVM on three combinations of different subsets of relevant variables: 1) PITA, miRanda and TargetScan scores on 3’utr region; 2) PITA, miRanda and TargetScan scores on coding region; 3) all of the variables considered in points 1 and 2.

**Fig. 2:**
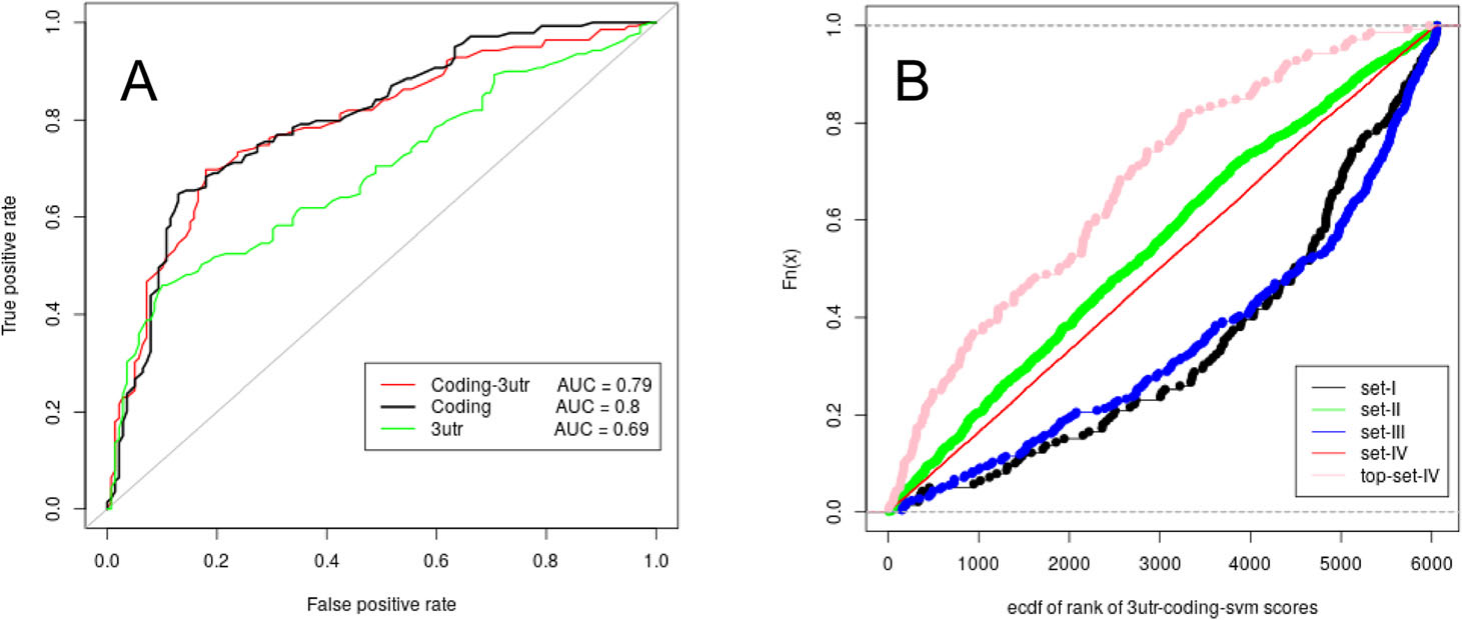
Overview of SVM prediction outcome. The SVM is trained with set I as positive set and top-set-IV as negative set. **a** shown ROC curves are the result of a LOOCV analysis. PITA, miRanda and Targetscan scores related to 3’utr (green line), coding region (black line) and both (red line) are user to train the SVM. **b** ECDF of the rank of ComiR scores obtained for the genes of set I (black), set II (green), set III (blue), set IV (red) and top-set-IV (pink)

Figure 2a compares the ROC curves obtained from a LOOCV analysis. The SVM trained on the coding region features has a higher predictions capacity than the SVM on 3’utr region features (coding vs 3’utr, *p*-value = 0.0005). On the other hand, the joint use of 3’utr and coding region information doesn’t significantly improve the performance (coding vs coding+ 3’utr; p-value = 0.31). In Fig. 2b we compare the empirical cumulative distribution functions (ECDF) of the rank of ComiR scores obtained for the genes in the four sets of the dataset with the coding+ 3’utr model. Similar results are obtained for the predictions obtained with the 3’utr only model and the coding region only model. We observe that both set I and set III show significantly higher ComiR scores than the whole dataset scores (*p*-value = 10e-12 and 10e-18 respectively). On the contrary, set II doesn’t show significantly higher ComiR scores than the whole dataset.

**Fig. 3:**
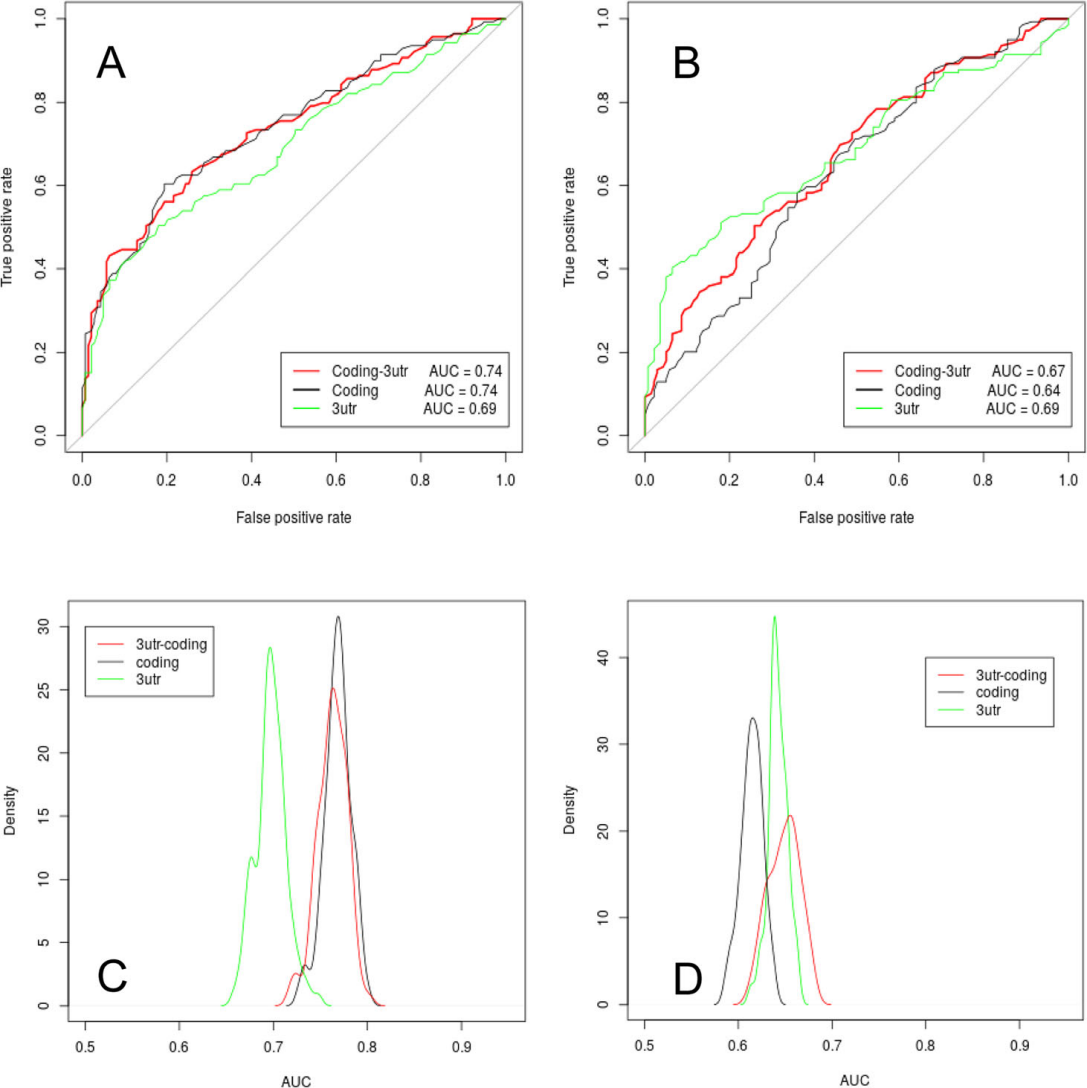
Overview SVM performance when using set I as training positive set and set III as positive testing set and vice versa. **a** ROC analysis results obtained by using set I and top-set-IV as training set and one example of ran-set-III and top2-set-IV as test set; **b** ROC analysis results obtained by using one example of ran-set-III and top2-set-IV as training set and set I and top-set-IV as test set; **c** AUC values distribution of 100 ROC analysis as described in **a** associated with different ran-set-III sampling. **c** AUC values distribution of 100 ROC analysis as described in **b** associated with different ran-set-III sampling

To further explore the behaviour of the sets I and III, and the performance of the SVM, we performed ROC analyses by alternatively using the two sets as training and testing set. Specifically, to obtain comparable AUC values, we randomly selected 139 genes from set III (ran-set-III) and the 139 most highly expressed genes (named top2-set-IV) after the first 139 included in the top-set-IV set. Figure 3a shows the ROC analysis results obtained by using set I and top-set-IV as training set and one of the ran-set-III and top2-set-IV as test set. The described training and testing set were then switched and the ROC analysis results are shown in Fig. 3b. We performed 100 of such tests, each time by randomly selecting a different ran-set-III set, and the distribution of the obtained AUC values is reported in Fig. 3c-d. In this case, we keep obtaining acceptable AUC values, in the range [0.6–0.8]. The lower AUC values, as compared to Fig. 2a, are due to the fact that the training and testing sets are selected from two different pools of genes and it is evident that a better efficiency is obtained when set I, instead than set III, is used as positive training set.

**Fig. 4:**
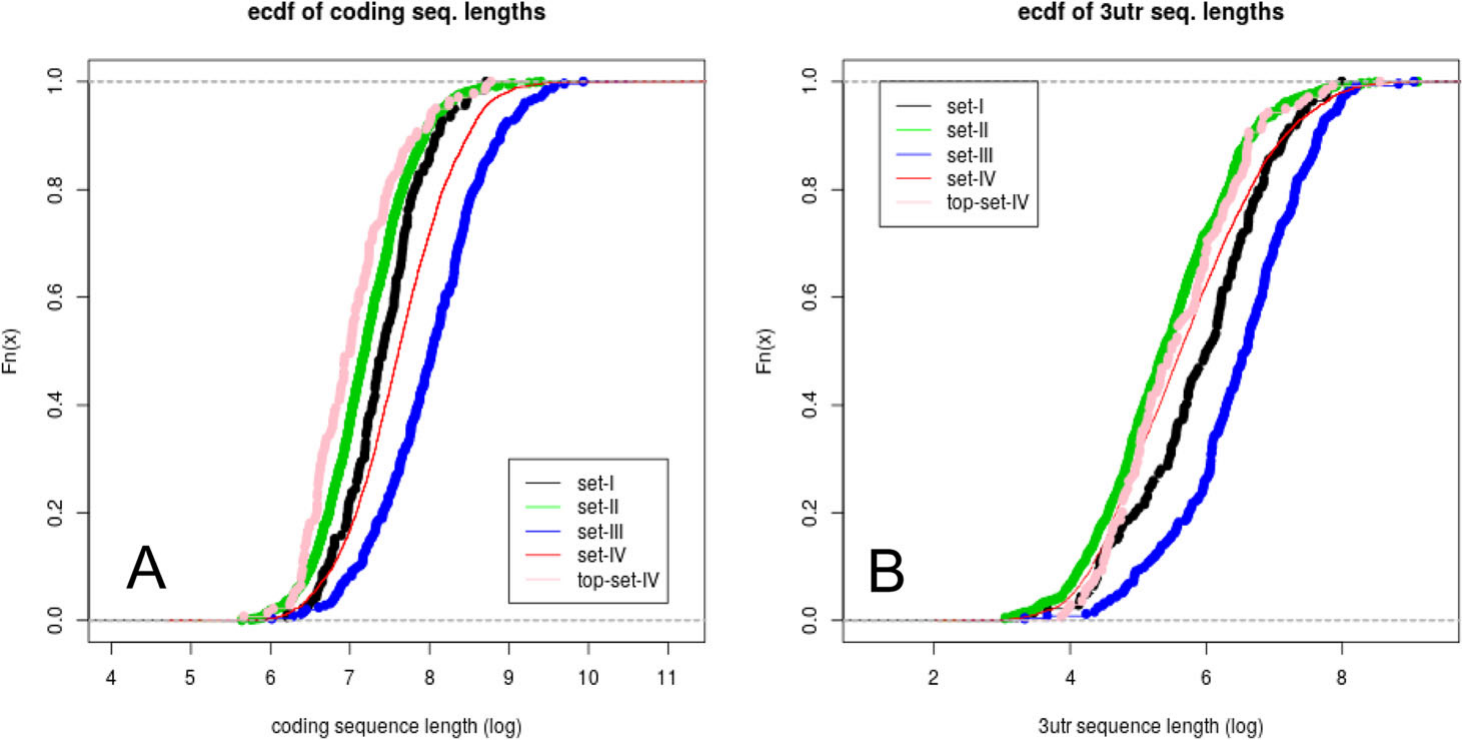
Overview of gene sequences lengths. **a** ECDF of 3’utr sequence lengths, **b** ECDF of coding region sequence lengths in the analyzed sets of genes

**Fig. 5:**
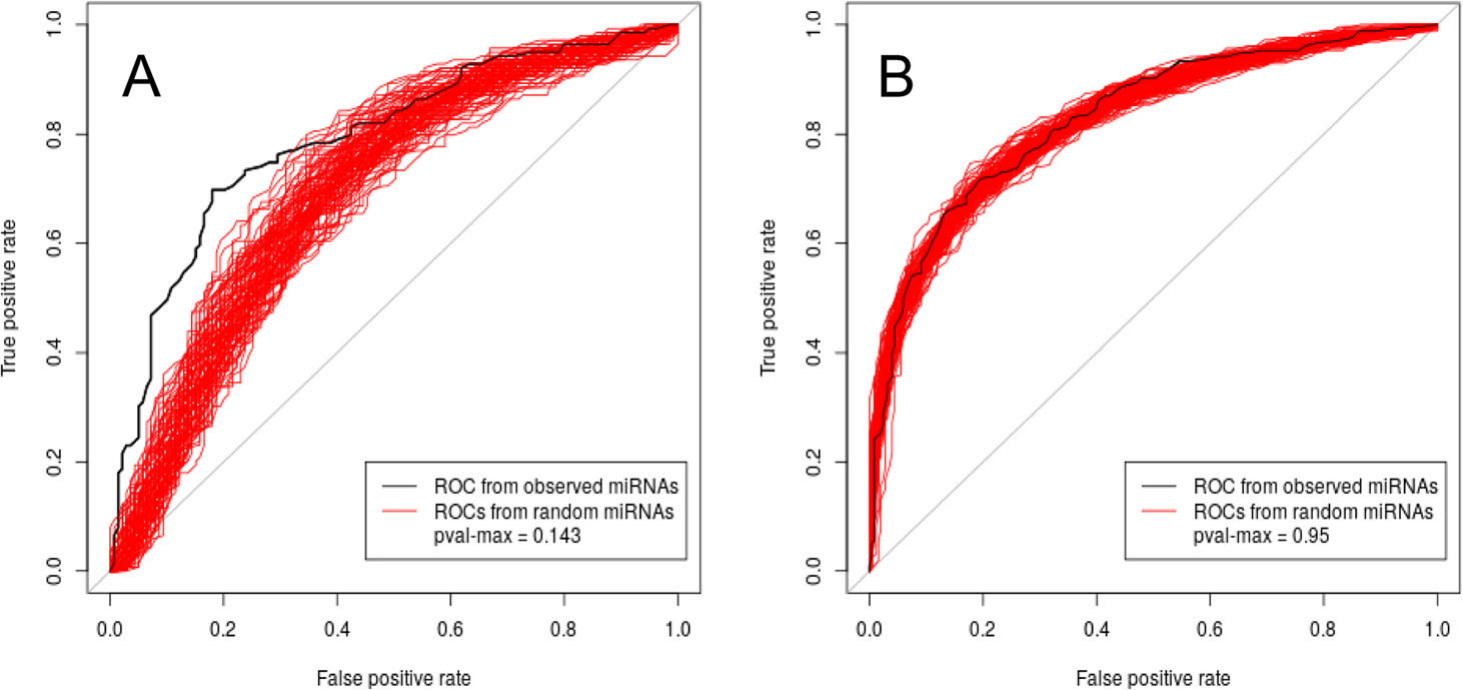
Overview of SVM performance with simulated miRNA expression profiles. The black line is associated to LOOCV test result obtained with the original miRNA expression profile, the red lined with the simulated profiles. In **a** we used set I as positive training set, in **b** we used set III

Figure 4 shows the ECDF of the sequence lengths of the analyzed sets. Genes of set III have significantly higher 3’utr and coding region lengths. Due to the additive calculation of the scores used to feed the SVM, it is expected that the length of the sequence plays a role in distinguishing the targets. To detect whether the SVM predictions are significantly dependent by the used miRNA expression profile, we performed a set of 100 LOOCV tests, each one performed by using a simulated miRNA expression profile to compute the training dataset. Specifically, each simulated miRNA expression profile was obtained by associating the original 28 expression values with a set of 28 randomly selected miRNAs (among the 469 Dme miRNAs). Figure 5a shows the ROC analysis results obtained with the simulated profiles (red lines) in comparison with the original profile (black line). It is evident that the performance in predicting the targets is significantly higher when the scores used to train the SVM are computed with the original miRNA expression profile. This effect is less evident when the set III is used as positive set (Fig. 5b), probably due to the fact that set III is strongly characterized by long RNA sequences and this feature is predominant in the training.

**Fig. 6:**
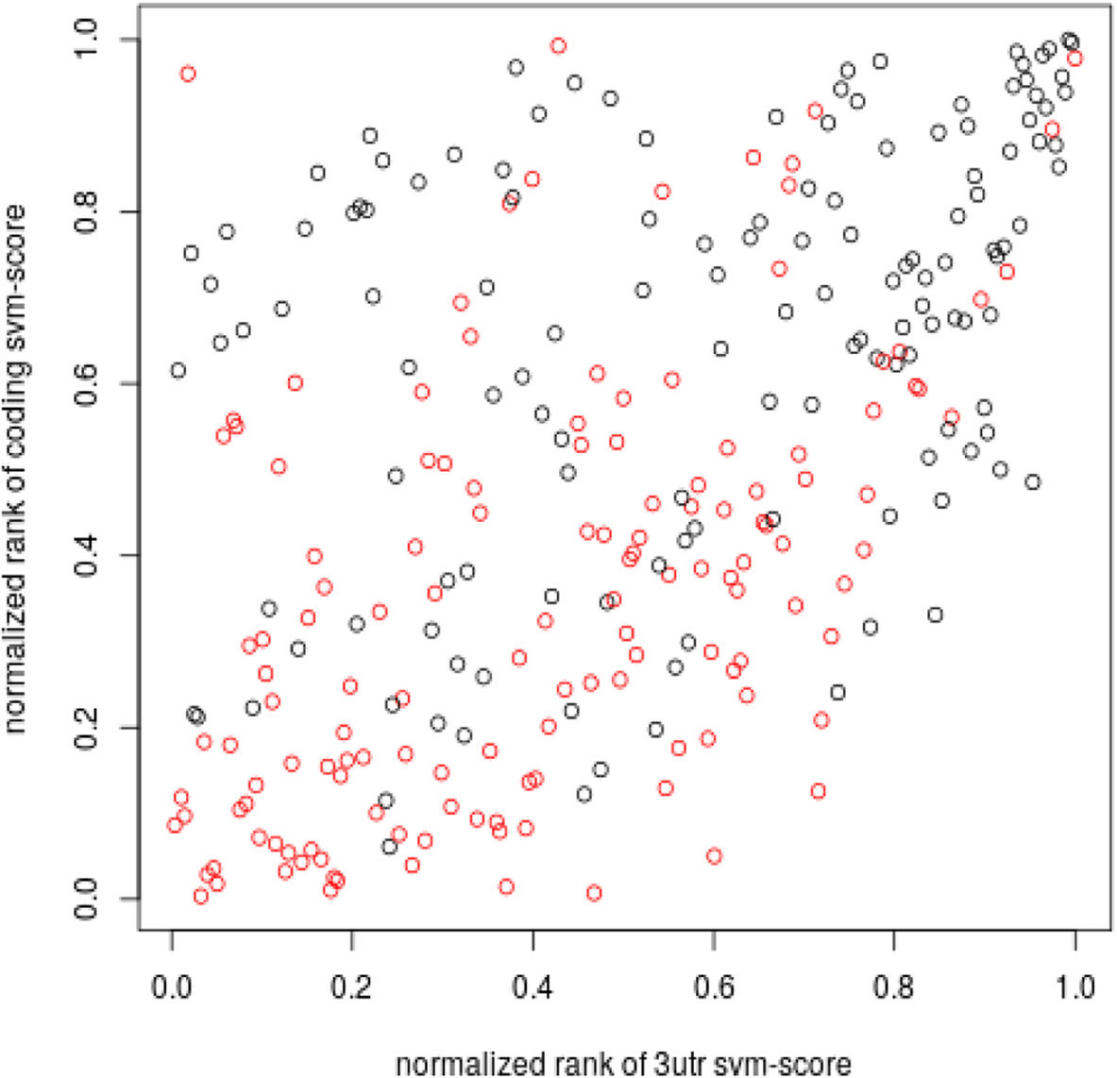
Scatter plot of SVM scores obtained with coding region based model vs 3’utr based model. The SVM is trained with set I as positive set and top-set-IV as negative set. Black points refer to the positive set, red points to the negative set

Training the SVM with both the 3’utr and coding region information doesn’t produce an improvement in the prediction efficiency. Figure 6 shows the scatter plot of the 3’utr-based predictions rank vs the coding region-based predictions rank of the positive and negative sets. It is evident that the two SVM models trained with 3’utr or coding region information separately, prioritize differently the genes, and neither of the two models should be discarded.

## Discussion

The presence of miRNA binding sites in the coding region of the genes has been already described in the scientific literature [19], although it is less explored than the association of miRNAs with the 3’utr. As mentioned already, the current version of ComiR only considers the binding sites predicted within the 3′ untranslated region. To fill such a gap of information, we decided to use the binding sites predicted in the coding region. Coding regions are significantly longer than the 3’utr, and the computational effort needed to predict their binding sites is probably one of the reasons why target-prediction tools are not extensively applied to them. The main objective of the paper is therefore to test whether adding the binding sites on the coding regions improves the miRNA target prediction.

If we compare the old version of ComiR results [8] and the results obtained here by using the 3’utr region only model, we noticed a significant drop in the performance of the upgraded version with respect to the first version of ComiR. We attributed this drop to the missing use of the mirSVR predictions and to the whole upgrade to the current release of the 3’utr sequences used to run the used miRNA target prediction tools, which changed significantly the efficiency in predictions of each single tool. Nevertheless, it is desirable to ensure the maintenance of the algorithm by upgrading the predictions database with the most recent sequences releases. Our results show that, focusing on the results obtained with the current sequences releases, the ComiR algorithm is significantly improved by considering the binding sites predicted in the coding region, outperforming the efficiency obtained by the algorithm when using only the 3’utr binding sites.

We observe that combining the information of both 3’utr and coding region binding sites in the SVM model doesn’t improve the performance of the prediction algorithm. This result is not due to a redundancy in 3’utr and coding region information. In fact, using the information carried by the binding sites presence in 3’UTR and coding region separately leads to the prediction of different sets of genes, both showing a significant enrichment of the positive training set. Our conclusion is that both the trained SVMs should be utilized to obtain a complete vision of the target prediction, and further analysis will be conducted to unravel the peculiarities of the two different predicted sets.

Our results suggest that ComiR scores prioritize the targets that are functionally degraded (set I and set III), while genes that are co-immunoprecipitated with the RISC protein AGO1 are not significantly predicted (set II). In addition, training the SVM with set II as positive set, generates a SVM model that doesn’t predict efficiently the set I training set (data not shown). On the other hand, set III genes show significantly longer 3’utr and coding region lengths, and this peculiarity could be the main reason for its good performance as positive set. We confirm to consider the set I as the most trustable positive set, because these genes are confirmed by two independent experimental approaches, whereas set II and III contain genes that have been detected by only one experimental approach each. The asymmetry in the response and the characteristics of these two sets of genes lead to the observation that both the experimental approaches, i.e. the RISC machinery proteins inhibition and immunoprecipitation, should be applied to detect a valid miRNA target set.

## Conclusion

Our results indicate that binding sites predicted in the genes coding region are valuable information in order to efficiently predict the functional targets of a set of miRNAs by their integration in the ComiR algorithm framework. We currently aim at finding the best way to combine the two scores obtained by training the SVM with the 3’UTR and the coding region separately. Further analysis will be conducted to analyze data from other species, by using positive and negative set of miRNA targets obtained through the comparison of results from both RISC proteins inhibition and immunoprecipitation.

## Data Availability

The datasets analyzed during the current study are available in supporting information of [14], ST1 - miRNA expression profile, ST8 - gene sets composition.

## Abbreviations

Dme: *Drosophila melanogaster*
ECDF: Empirical Cumulative Distribution Function
LOOCV: Leave One Out Cross Validation
RISC: RNA Induced Silencing Complex
SVM: Support Vector Machine
